# Radiological features of pancreatic desmoid-type fibromatosis: a case series and systematic review

**DOI:** 10.1007/s00261-024-04570-8

**Published:** 2024-09-15

**Authors:** Tomoya Tanishima, Ryo Kurokawa, Miyuki Sone, Yudai Nakai, Masahiko Kusumoto

**Affiliations:** 1 Department of Diagnostic Radiology, National Cancer Center Hospital, 5-1-1 Tsukiji, Chuo-ku, Tokyo, 104-0045 Japan; 2 Department of Radiology, Graduate School of Medicine, The University of Tokyo, 7-3-1 Hongo, Bunkyo-ku, Tokyo, 113-8655 Japan

**Keywords:** Desmoid, Pancreatic tumor, Systematic review, Computed tomography, Magnetic resonance imaging

## Abstract

**Purpose:**

This retrospective study aimed to investigate the radiological features of pancreatic desmoid-type fibromatosis (PDF) and systematically review the previous publications and two new cases.

**Methods:**

We searched PubMed, Cochrane Library, and Web of Science Core Collection and included 31 patients with pathologically proven PDFs with analyzable preoperative computed tomography (CT) and magnetic resonance imaging, including two patients from our institution and 29 patients from 28 publications. Two board-certified radiologists reviewed all images.

**Results:**

The median age of the patients was 39 years, with a male dominance observed (male, 54.8% vs. female, 45.2%). Abdominal pain was the most frequent symptom, occurring in 58.1% of cases. Surgical resection was performed in all cases of PDFs, resulting in a recurrence rate of 8.3% (2/24). The tumors were most commonly located in the pancreatic tail (23/31, 74.2%). In terms of morphology, a “solid” shape was most prevalent (14/31, 45.2%), followed by a “solid and cystic” shape (9/31, 29.0%) and a “cystic” shape (8/31, 25.8%). Characteristic radiological features included heterogeneous enhancement of the solid portion of the tumors on CT scans (13/20, 65%), moderate-to-weak enhancement in the late phase on CT (16/17, 94.1%), and a presence of cystic components in the tumors (17/31, 54.8%). In 16.1% (5/31) of PDFs, the cystic component was pathologically confirmed to be a dilated pancreatic duct.

**Conclusion:**

We summarized the clinical and imaging characteristics of PDF. Although the incidence may not be high, cystic components suggesting a dilated pancreatic duct within the tumor are unique imaging features in PDF.

## Introduction

Desmoid-type fibromatosis, also known as desmoid tumor or aggressive fibromatosis, are locally aggressive but non-metastasizing deep-seated (myo)fibroblastic neoplasms with infiltrative growth and propensity for local recurrence [1]. Desmoid-type fibromatosis are estimated to affect 2–4 patients per 1 million population per year [2]. Patients tend to be young, with a median age of 37–39 years [2–4]. Most desmoid-type fibromatosis occur sporadically; however, they may also occur in association with the hereditary syndrome such as familial adenomatous polyposis (FAP) [5]. In a previous study, tumor locations were as follows: extremity (42%), abdominal or retroperitoneal cavity (20%), abdominal wall (17%), and chest wall (15%) [3]. Among desmoid-type fibromatosis occurring in the abdominal cavity, the small bowel mesentery is the most common site [2]. Desmoid-type fibromatosis have rarely been reported to involve the pancreas, with 42 cases reported in the literature, typically as single cases [6]. Moreover, surgical resection is performed for 85.7% of pancreatic desmoid-type fibromatosis (PDF), with a recurrence rate of 4.8% [6], which is lower than that for desmoid-type fibromatosis (44.3%) [3].

While the majority of literature concerning PDF has focused on its pathological, histological, and clinical findings, knowledge of the radiological imaging findings of this tumor has been limited. To the best of our knowledge, this is the most comprehensive systematic review to date on the radiological features of PDF. The purpose of this study was to summarize the demographic and clinical data of patients and the imaging findings of PDF to identify features that are helpful in diagnosis.

## Material and methods

### Study selection

We searched PubMed, the Cochrane Library, and the Web of Science Core Collection using the following search terms on September 3, 2023, without any language or date limits:

- (“fibromatosis, aggressive” [mh] OR “fibroma” [mh] OR desmoid*[tiab] OR fibromatosis[tiab] OR fibroma[tiab]) AND (pancreas[mh] OR “pancreatic diseases” [mh] OR pancreas[tiab] OR pancreatic[tiab]) AND (“diagnostic imaging” [sh] OR diagnostic imaging[mh] OR radiologic*[tiab] OR radiology[tiab] OR imaging[tiab] OR “magnetic resonance” [tiab] OR “computed tomography” [tiab] OR “computerized tomography” [tiab] OR MRI[tiab] OR CT[tiab]) for PubMed;
- ([mh “fibromatosis, aggressive”] OR [mh “fibroma”] OR (desmoid* OR fibromatosis OR fibroma):ti,ab,kw) and ([mh pancreas] OR [mh “pancreatic diseases”] OR (pancreas OR pancreatic):ti,ab,kw) for Cochrane Library;
- (fibroma OR desmoid* OR fibromatosis OR fibromatoses) AND (pancreas OR pancreatic) to the Web of Science Core Collection.

Publications were considered eligible if they included all of the following criteria:

The inclusion criteria were as follows:

- The content was relevant to PDF;
- Either a magnetic resonance imaging (MRI) or computed tomography (CT) image of the tumor was available;

The exclusion criteria were as follows:

- Duplicate cases;
- The full text was unavailable.

We obtained institutional review board exemption for including two unpublished cases with pathologically proven PDF and their preoperative CT or MRI findings obtained at our hospital. Data were acquired in compliance with all applicable regulations of the Health Insurance Portability and Accountability Act.

This study was performed according to the Preferred Reporting Items for Systematic Reviews and Meta-Analyses (PRISMA) 2020 statement [7].

### Data analyses

Two board-certified radiologists independently reviewed all studies and the CT and MR images of the eligible examinations. When discrepancies arose between the two reviewers, another board-certified radiologist made a tie-breaker decision.

### Collected data

The following data were collected:

Demographics

- Patient age
- Sex

Clinical

- Presenting complaint
- Recurrence after treatment (presence or absence)
- Survival outcomes (survive or dead)
- Follow-up duration (month since diagnosis)

Radiological

- Tumor size and location
- Tumor margin status (well-or ill-defined)
- Tumor morphology (solid, cystic, or solid and cystic)
- CT attenuation (high density: higher than muscles; intermediate density: between cerebrospinal fluid [CSF] and muscles; low density: lower than CSF)
- CT enhancement of the solid portion (homogeneous or heterogeneous; strong: stronger than the pancreas; moderate: almost the same as the pancreas; weak: weaker than the pancreas).
- T2-weighted image (T2WI) signal intensity (high intensity, higher than fat; intermediate intensity, between fat and muscles; low intensity, lower than muscles)
- Fat-suppressed (fs) T2WI signal intensity (high intensity: higher than CSF; intermediate intensity: between CSF and muscle; low intensity: lower than muscle)
- T1-weighted image (T1WI) and fsT1WI signal intensities (high intensity: higher than muscles; intermediate intensity: between muscles and CSF; low intensity: lower than CSF)
- The presence or absence of hemorrhage
- MRI enhancement of the solid portion (homogeneous or heterogeneous; strong, stronger than the pancreas; moderate, almost the same as the pancreas; weak, weaker than the pancreas)
- The presence or absence of diffusion restriction
- The maximum standardized uptake value on F-18 fluorodeoxyglucose uptake on positron emission tomography (PET)/CT

### Quality assessment

For a systematic review of case-based studies, we used a tool proposed by Murad et al. to evaluate the methodological quality of case reports/series [8]. This tool is based on the eight signaling questions in four domains (selection, ascertainment, causality, and reporting) and has been widely used in previous studies [9–12].

## Results

### Study selection

**Fig. 1:**
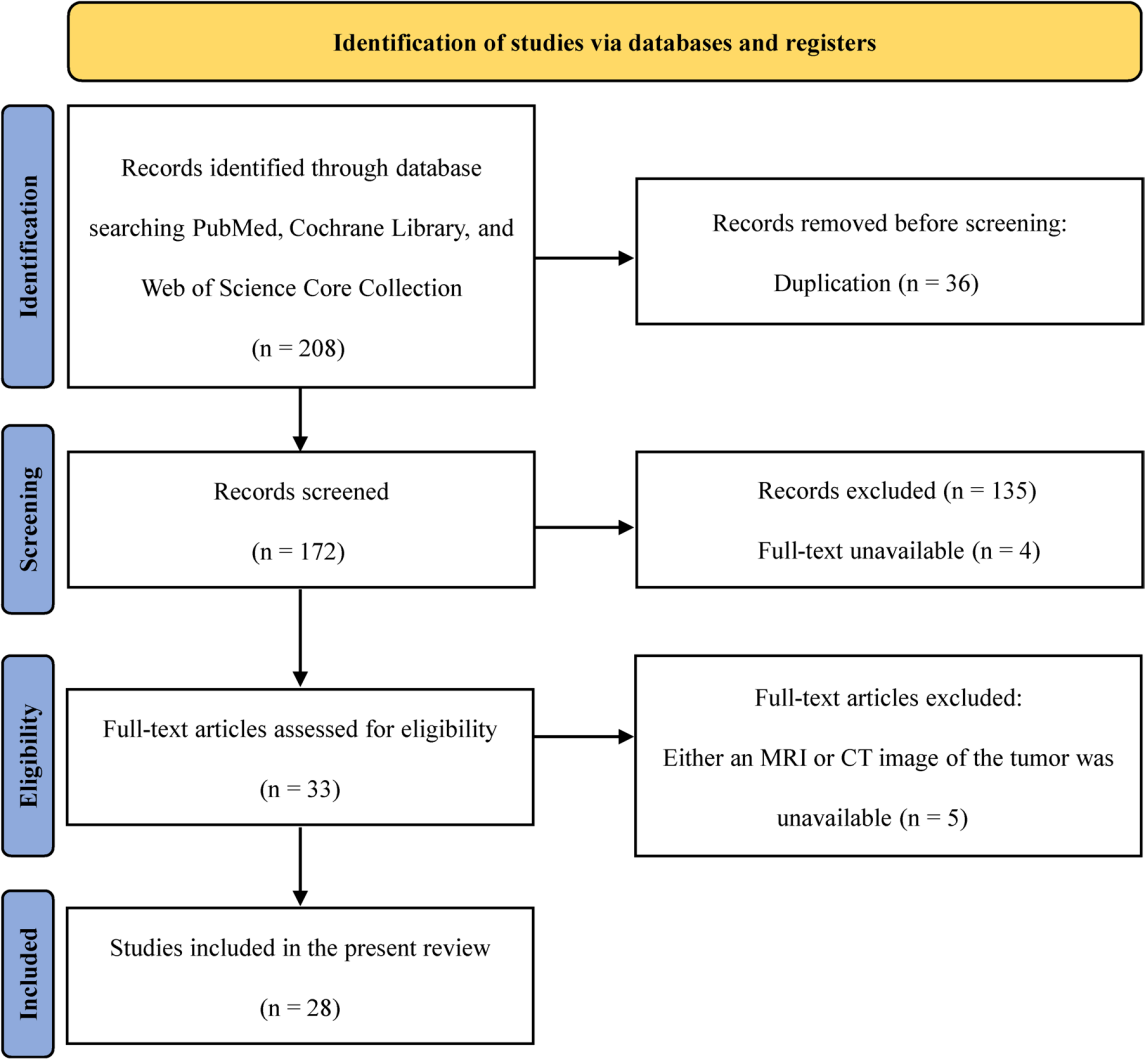
Flow diagram of study identification

A total of 208 abstracts were screened according to the PRISMA 2020 guidelines [7], of which 39 potentially eligible studies were assessed for review. After excluding 11 studies, 28 studies with 29 cases met the criteria for the systematic review [6, 13–39]. The identification process used in these studies is summarized in Fig. 1. The publication year of the included studies ranged from 1996 to 2023. We added two unpublished cases from our hospital (Table 1), resulting in a final study cohort of 31 patients who underwent PDF.

**Table 1:** Demographic, clinical, and radiological data of two patients with pancreatic desmoid-type fibromatosis in our hospital

Demographic & Clinical data	Patients		1	2
	Age (years)		20	22
	Sex		Female	Female
	Abdominal pain		Yes	Yes
	Family history of FAP		No	No
	History of abdominal trauma or surgery		No	No
	Surgery for PDF		Yes	Yes
	Chemotherapy or radiation for PDF		No	No
	Recurrence		No	No
	Current status at the end of 2023		Survive	Survive
	Follow up duration (month)		18	174
Radiological data	Tumor size (mm)		284	54
	Tumor site		Tail	Tail
	Tumor margin		Well-defined	Ill-defined
	Morphology		Solid and cystic	Solid and cystic
	Solid portion	CT attenuation	Intermediate	Intermediate
		CT enhancement	Homogeneous	Homogeneous
		In the arterial phase	Weak	Weak
		In the late phase	Weak	Moderate
		T2WI	Intermediate	Intermediate
		Fat-suppressed T2WI	High	
		T1WI	High	Intermediate
		Fat-suppressed T1WI	High	
		MRI enhancement	Homogeneous	Homogeneous
		In the arterial phase		Weak
		In the late phase	Strong	Strong
		Diffusion restriction	No	
	Cystic portion	CT attenuation	Low	Low
		T2WI	High	High
		Fat-suppressed T2WI	High	High
		T1WI	Intermediate	Low
		Fat-suppressed T1WI	High	
	Hemorrhage		Yes	No
	Metastasis		No	No

Gray cell: not available, *FAP* familial adenomatous polyposis, *PDF* pancreatic desmoid-type fibromatosis, *CT* computed tomography, *T2WI* T2-weighted imaging, *T1WI* T1-weighted imaging, *MRI* magnetic resonance imaging

### Risk of bias assessment

As we extracted data from case-based studies, where the selection method was rarely mentioned, a selection bias may have been introduced. Tumor size was available for 30 of the 31 cases (96.7%). Treatment strategies and outcomes were ascertained for 27 of 31 (87.1%) and 23 of 31 (74.2%) patients, respectively. The follow-up duration of the patients ranged from 2 months to 5 years. Because we evaluated cases using preoperative CT or MR, our research can be replicated by other investigators.

### Demographic and clinical data

The demographic and clinical data of the 31 patients are summarized in Table 2. The median age at diagnosis was 39 years (range, 13–75 years), with no evidence of differences in the incidence by age from 10-to 70-year-olds. More male patients (17/31, 54.8%) were affected than were female patients (14/31, 45.2%). A total of 5.0% (1/20) and 33.3% (7/21) of the patients had a family history of FAP and a history of abdominal surgery, respectively.

**Table 2:** Demographic and clinical information of the 31 patients with pancreatic desmoid-type fibromatosis

Demographic		
Median age at diagnosis (years [range]) (n = 31)	39 [13–75]	
Sex	Male = 17, Female = 14	
Family history of FAP	1/20 (5.0%)	
History of abdominal trauma	0/9 (0%)	
History of abdominal surgery	7/21 (33.3%)	
Clinical		
Abdominal pain	18/31 (58.1%)	
No symptoms	7/31 (22.6%)	
Nausea	6/31 (19.4%)	
Vomiting	6/31 (19.4%)	
Abdominal bloating	5/31 (16.1%)	
Anorexia	3/31 (9.7%)	
Weight loss	3/31 (9.7%)	
Back pain	3/31 (9.7%)	
Fever	2/31 (6.5%)	
Chest pain	1/31 (3.2%)	
Diarrhea	1/31 (3.2%)	
Constipation	1/31 (3.2%)	
Melena	1/31 (3.2%)	
Dysphasia	1/31 (3.2%)	
Initial treatment strategy (n = 27)		
Surgery alone	26/27 (96.3%)	
Surgery and COX-2 inhibitor	1/27 (3.7%)	
Recurrence after surgery	2/24 (8.3%)	
Patient status	Survive = 24/25 (96.0%), Death = 1/25 (4.0%)	
Follow up duration (median [range]) (n = 24)	19 months [2–174]	
Pathological		
Pancreatic duct dilatation	5/31 (16.1%)	
IHC	Positive	Negative
Nuclear β-catenin (n = 24)	24	0
α-SMA (n = 16)	12	4
CD34 (n = 15)	2	13
CD117 (n = 14)	1	13
Desmin (n = 13)	1	12
S100 (n = 13)	1	12
Vimentin (n = 5)	5	0
ALK (n = 5)	0	5
Ki67 (n = 4)	0	4
DOG1 (n = 4)	0	4
Pancytokeratin (n = 4)	0	4
Calretinin (n = 3)	0	3
EMA (n = 2)	0	2
Caldesmon (n = 2)	0	2
MUC4 (n = 2)	0	2
WT1 (n = 2)	0	2
ER (n = 2)	0	2
STAT6 (n = 2)	0	2
PgR (n = 1)	0	1
CD10 (n = 1)	1	0
Bc12 (n = 1)	0	1
Cytokeratin (n = 1)	1	0
D2-40 (n = 1)	0	1
MC (n = 1)	0	1
MDM2 (n = 1)	0	1
CDK4 (n = 1)	0	1
CK5/6 (n = 1)	0	1
Actin (n = 1)	1	0
Sox10 (n = 1)	0	1

*FAP* Familial adenomatous polyposis, *COX-2* cyclooxygenase-2, *IHC* immunohistochemistry

The most frequent chief complaint was abdominal pain (18/31, 58.1%), followed by absence of symptoms (7/31, 22.6%). Surgery alone (26/27, 96.3%) was the most commonly used treatment strategy, and the incidence of tumor recurrence after complete resection was low (2/24, 8.3%). In 16.1% (5/31) of the PDFs, the cystic component was pathologically confirmed to be a dilated pancreatic duct. Moreover, 24 cases of PDFs showed nuclear staining for β-catenin. Other immunohistochemical results for the PDFs are summarized in Table 2.

### Radiological findings

Radiological findings are summarized in Table 3. The median tumor size was 65.5 mm (range: 19–284 mm). The most frequent tumor site was the tail of the pancreas (23/31, 74.2%), followed by the head (4/31,12.9%) and body (4/31,12.9%). Solid shape (14/31, 45.2%) was the most frequent morphology, followed by solid and cystic shapes (9/31, 29.0%) and cystic shape (8/31, 25.8%). The tumor margins were ill-defined (17/31, 54.8%) more often than were well-defined (14/31, 45.2%). The solid portion was most likely to show intermediate density on CT (5/5, 100%), weak enhancement in the arterial phase on CT (9/9, 100%), moderate-to-weak enhancement in the late phase on CT (16/17, 94.1%), intermediate-to-high intensity on T2WI/fsT2WI (4/4, 100%), intermediate-to-high intensity on T1WI (3/3, 100%) /fat-suppressed T1WI (2/2, 100%) and non-restricted diffusion (1/2, 50%). The enhancement patterns of the solid portion on MRI varied among patients. The cystic portion showed intermediate-to-low density on CT (5/5, 100%), high intensity on T2WI/fat-suppressed T2WI (2/2, 100%), intermediate-to-high intensity on T1WI (3/3, 100%), and high intensity on fat-suppressed T1WI (1/1, 100%). Hemorrhage was observed in a limited number of patients (1/31, 3.2%). 18F-FDG-PET/CT images were available in only two cases, but avid FDG uptake was observed in both cases. None of the patients in our study had tumor involvement at any other site. Additionally, none of the cases exhibited dilation of the common bile duct or intrahepatic bile ducts, nor was there any evidence of lymph node enlargement. We observed no apparent differences in the imaging findings between FAP-associated and non-FAP-associated PDF.

**Table 3:** Radiological characteristics of the 31 patients with pancreatic desmoid-type fibromatosis

Parameters	
Size (median [range]) (n = 30)	65.5 mm [19–284]
Location	
Head	4/31 (12.9%)
Body	4/31 (12.9%)
Tail	23/31 (74.2%)
Morphology	
Solid	14/31 (45.2%)
Cystic	8/31 (25.8%)
Solid and cystic	9/31 (29.0%)
Tumor margin	
Well-defined	14/31 (45.2%)
Ill-defined	17/31 (54.8%)
CT attenuation of solid portion	
Intermediate	5/5 (100%)
CT attenuation of cystic portion	
Intermediate	1/5 (20%)
Low	4/5 (80%)
CT enhancement of solid portion	
Homogeneous	7/20 (35%)
Heterogeneous	13/20 (65%)
In the arterial phase	
Weak	9/9 (100%)
In the late phase	
Strong	1/17 (5.9%)
Moderate	8/17 (47.1%)
Weak	8/17 (47.1%)
T2WI signal intensity of solid portion	
High	2/4 (50%)
Intermediate	2/4 (50%)
Fat-suppressed T2WI signal intensity of solid portion	
High	1/4 (25%)
Intermediate	3/4 (75%)
T2WI signal intensity of cystic portion	
High	2/2 (100%)
Fat-suppressed T2WI signal intensity of cystic portion	
High	2/2 (100%)
T1WI signal intensity of solid portion	
High	1/3 (33.3%)
Intermediate	2/3 (66.7%)
Fat-suppressed T1WI signal intensity of solid portion	
High	1/2 (50%)
Intermediate	1/2 (50%)
T1WI signal intensity of cystic portion	
Intermediate	1/2 (50%)
Low	1/2 (50%)
Fat-suppressed T1WI signal intensity of cystic portion	
High	1/1 (100%)
Hemorrhage	1/31 (3.2%)
MRI enhancement of solid portion	
Homogeneous	2/3 (66.7%)
Heterogeneous	1/3 (33.3%)
In the arterial phase	
Weak	1/1 (100%)
In the late phase	
Strong	2/3 (66.7%)
Weak	1/3 (33.3%)
Diffusion restriction	1/2 (50%)
FDG uptake on PET/CT	
Avid	2/2 (100%)
No metastasis	7/7 (100%)

*CT* Computed tomography, *T2WI* T2-weighted imaging, *T1WI* T1-weighted imaging, *MRI* Magnetic resonance imaging, *FDG* F-18 fluorodeoxyglucose, *PET* Positron emission tomography

### Histological and immunohistochemical findings of our two cases

**Fig. 2:**
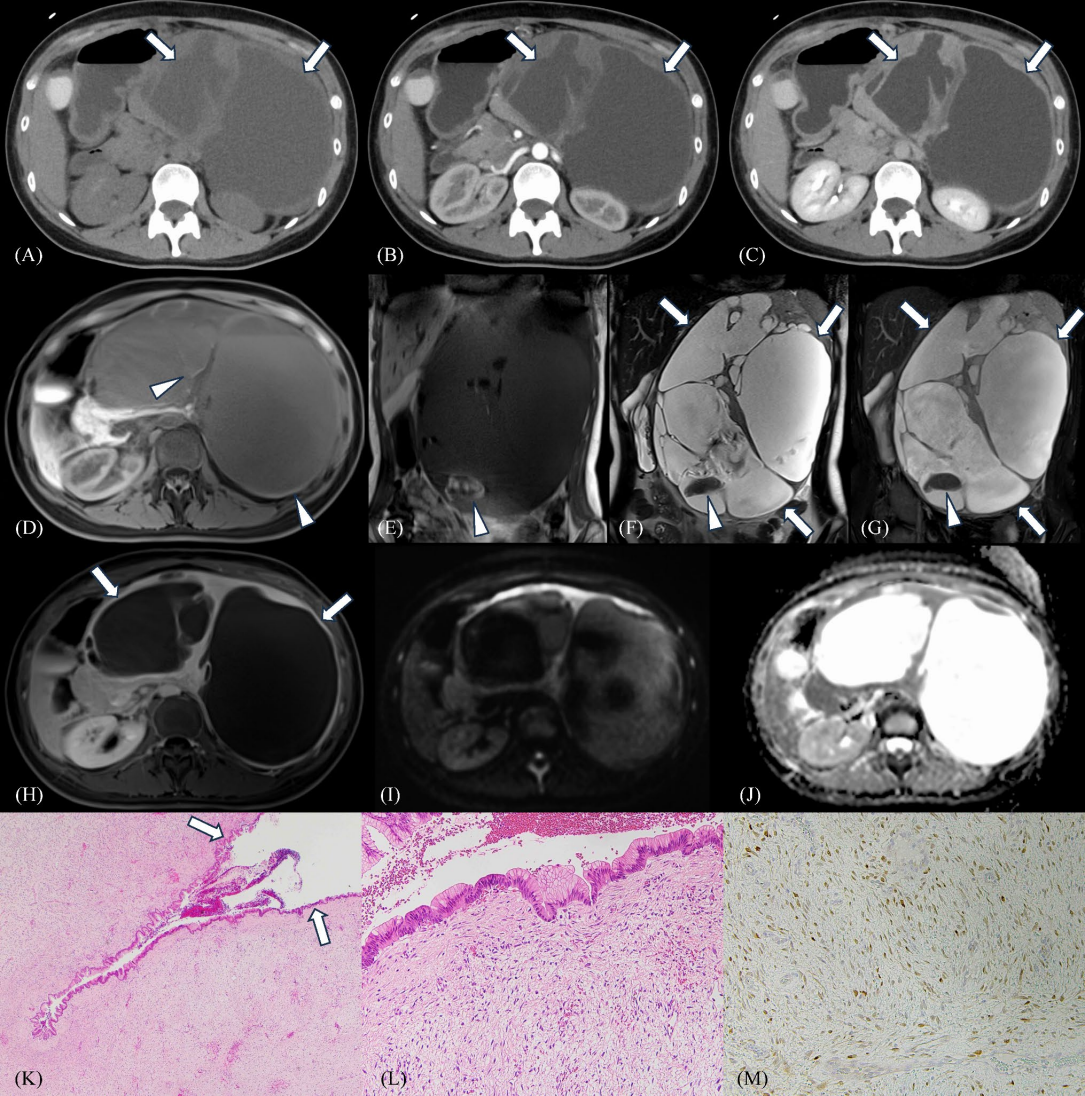
Pancreatic desmoid-type fibromatosis in a 22-year-old female presenting with abdominal bloating, constipation, and abdominal pain (case 1). CT shows a 27-cm well-delineated tumor with a solid and cystic morphology (A, plain; B, arterial-phase; C, delayed-phase). The solid portion shows delayed enhancement on contrast-enhanced CT (A, B, C) and MRI (H), high intensity on fat-suppressed T1WI (D) and coronal T1WI (E), intermediate intensity on coronal T2WI (F), high intensity on fat-suppressed coronal T2WI (G), and homogeneous enhancement (H). The cystic portion shows high intensity on coronal T2WI (F) and fat-suppressed coronal T2WI (G), and high intensity on fat-suppressed T1WI (D) and coronal T1WI (E). Most of the cystic portions are continuous (white arrows), some with hemorrhage (white arrowheads). Diffusion restriction is not observed with the mean ADC value of the solid portion of 1.50 × 10−3mm2/s (I, J). Low-power magnification with hematoxylin and eosin stain shows desmoid-type fibromatosis with prominent pancreatic ductal dilation (K, white arrow). High-power magnification with hematoxylin and eosin stain shows pancreatic ductal epithelium showing reactive changes without evidence of dysplasia (L). Corresponding β-catenin nuclear positivity is observed (M). CT Computed tomography, MRI magnetic resonance imaging, T1WI T1-weighted imaging, T2WI T2-weighted imaging, ADC apparent diffusion coefficient

**Fig. 3:**
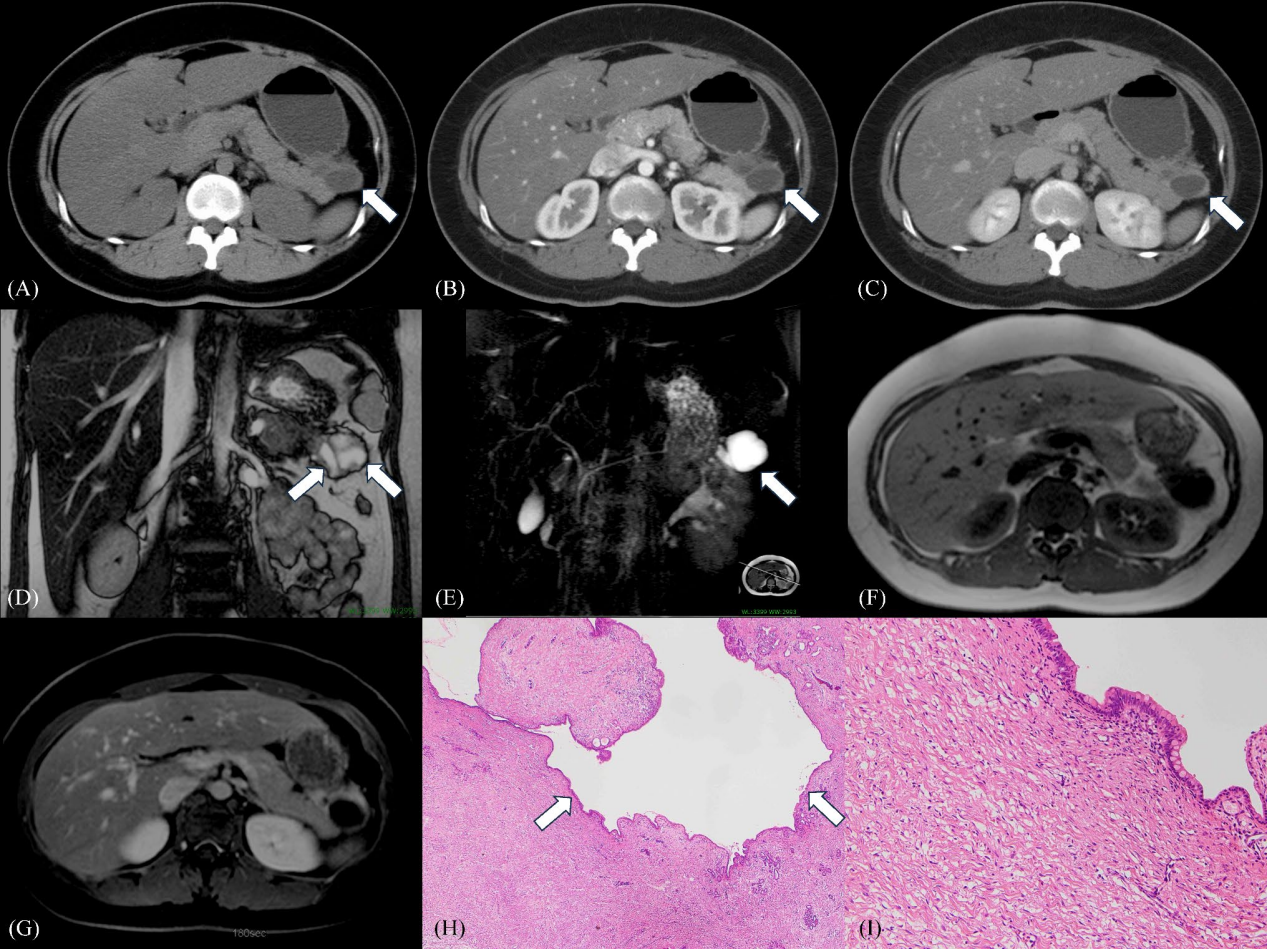
Pancreatic desmoid-type fibromatosis in a 20-year-old asymptomatic female (case 2). CT shows a 4-cm well-delineated tumor with a solid and cystic morphology (A, plain; B, arterial-phase; C, delayed-phase). The solid portion shows delayed enhancement on contrast-enhanced CT (A, B, C) and MRI (G), intermediate intensity on coronal T2 True Fast Imaging with Steady State Precession (True FISP) (D) and T1WI (F), and homogeneous enhancement (G). The cystic portion shows high intensity on coronal True FISP (D) and MRCP (E), and low intensity on T1WI (F). Most of the cystic portions are continuous (A–E, white arrows). Low-power magnification with hematoxylin and eosin stain shows desmoid-type fibromatosis with prominent pancreatic ductal dilation (H, white arrows). High-power magnification with hematoxylin and eosin stain shows pancreatic ductal epithelium showing reactive changes without evidence of dysplasia (I). Corresponding β-catenin nuclear positivity is observed (not shown). CT Computed tomography, MRI magnetic resonance imaging, T2WI T2-weighted imaging, T1WI T1-weighted imaging, MRCP magnetic resonance cholangiopancreatography

Surgical resection was performed in our two cases characterized by a cystic component on imaging examinations (Figs. 2 and 3). Most of the cystic portions were internally connected, forming a continuous structure rather than a cluster of separate cysts, as observed on CT and MRI examinations. In case 1 (Figs. 2), diffusion restriction was not observed with the mean apparent diffusion coefficient (ADC) value of the solid portion of 1.50 × 10⁻^3^ mm^2^/s. Histological sections from these two cases confirmed that all of the cystic component, observed in both tumors on imaging studies, corresponded to a dilated pancreatic duct. It was challenging to distinguish pathologically between the main pancreatic duct and the side branches in our two cases. In these cases, tumors infiltrating the pancreatic parenchyma around the dilated ducts were noted. For both, the pancreatic ductal epithelium exhibited reactive changes, with no evidence of dysplasia, and no findings suggestive of pancreatitis were observed. Immunohistochemical studies revealed that the tumor cells tested positive for beta-catenin, with nuclear staining, and negative for CD34, CD117, desmin, and S100.

## Discussion

In this study, we summarized the demographic, clinical, and radiological features of 29 patients with PDFs from 28 publications and two additional patients from our hospital. The median age of the patients was 39 years, with a male predominance (male, 54.8% vs. female, 45.2%) and abdominal pain being the most frequent symptom (58.1%). Surgical resection was performed in all PDFs, with a recurrence rate of 8.3% (2/24 patients). Tumors were most frequently observed in the pancreatic tail (23/31, 74.2%). Characteristic radiological features included heterogeneous enhancement of the solid portion of the tumors on CT (13/20, 65%), moderate-to-weak enhancement in the late phase (16/17, 94.1%), and the frequent presence of cystic components in tumors (17/31, 54.8%). To the best of our knowledge, this is the largest systematic review of radiological findings of PDFs.

Desmoid-type fibromatosis are rare neoplasms that occur sporadically or in the context of FAP. According to a study by Nieuwenhuis et al. [5], at least 7.5% of desmoid-type fibromatosis are associated with FAP. The calculated incidence of sporadic desmoid-type fibromatosis was 3.42 per million person-years, whereas, for the FAP population, the incidence was 2,784 per million person-years, indicating an over 800-fold increased risk of desmoid-type fibromatosis in patients with FAP. However, owing to the low incidence of FAP, most desmoid-type fibromatosis encountered in daily clinical practice are sporadic, as only 5% (1/20) of the patients had concomitant FAP in the present study.

Surgical resection remains the cornerstone of treatment for desmoid-type fibromatosis when R0 and function-sparing resection is achievable [40, 41]. However, in certain cases, achieving a resection with wide, clear margins to prevent tumor recurrence poses challenges due to the tumor’s locally invasive nature. In recent years, options such as observation and the use of anti-inflammatory drugs have emerged as potential treatment choices for the management of desmoid-type fibromatosis [41]. In the case of PDFs, there exists a reported case in which tumor control was achieved through partial cystectomy and administration of a cyclooxygenase-2 inhibitor [36]. Recurrence rates of desmoid-type fibromatosis have been reported to be 8% in cases associated with FAP and 12% in sporadic cases [5], and our study found that the recurrence rate of PDFs (8.3%) is generally consistent with that of previous studies.

Immunohistochemistry for specific cell markers of various origins is an effective tool for distinguishing PDF from other pancreatic tumors. PDF exhibits distinctive immunohistochemical features, primarily characterized by nuclear staining of β-catenin, which sets it apart from other mesenchymal tumors of pancreatic origin [42]. In this study, nuclear β-catenin staining was observed in 24 cases of PDFs. Other immunohistochemical results were also generally consistent with those of previous research [43]. Expression was negative for CD34, CD117, S-100, and desmin, which excluded a gastrointestinal stromal tumor, schwannoma, leiomyoma, and leiomyosarcoma, respectively [44].

A comprehensive understanding of the radiological findings of PDF has been lacking owing to the rarity of this condition. We found that the “solid” morphology was the most common, followed by the “solid and cystic” and “cystic” morphology. Interestingly, 29.4% (5/17) of the PDFs contained characteristic cystic components on imaging, and the cystic components were pathologically identified as pancreatic duct dilatation. We believe that the cystic component within the tumor, consisting of dilated pancreatic ducts, is a unique imaging and pathological finding of PDFs that is not observed in desmoid-type fibromatosis at other sites. Regarding the mechanism of pancreatic ductal dilatation, some reports suggest that it may be a secondary change associated with pancreatic ductal obstruction [6, 38]; however, in these two previously reported cases, pancreatic duct obstruction due to the tumor was not confirmed pathologically. In contrast, in our two cases, no findings suggestive of pancreatitis were observed. While Clarke-Brodber et al. also reported that none of the 42 patients who underwent PDFs showed clinical features of pancreatitis [6], suggesting that the mechanism of pancreatic ductal obstruction is unlikely, it is important to note that not all cases of pancreatic ductal adenocarcinoma (PDAC) present with pancreatitis despite having MPD dilatation. This suggests that the absence of pancreatitis does not entirely rule out ductal obstruction. Therefore, further studies are needed to clarify the exact mechanism of ductal dilatation in PDF, considering both mechanical obstruction and other potential factors. We speculated that the primary cause of pancreatic ductal dilatation in PDF is the disruption of the supporting tissue of the pancreatic ducts due to tumor infiltration rather than mechanical ductal obstruction by the tumors. Differential diagnosis includes pancreatic tumors such as neuroendocrine neoplasms, solid pseudopapillary neoplasms, serous cystic neoplasms, and mucinous cystic neoplasms [6]. When PDFs are considered in the differential diagnosis through imaging examinations, if PDFs are unresectable or resectable but asymptomatic, options such as observation or biopsy, in the case of solid tumors, shoud be considered. Even when surgery is performed on symptomatic and resectable PDFs, it is essential to consider PDFs in the differential diagnosis when characteristic imaging findings are observed, as their local invasiveness and high recurrence rate significantly influence decisions regarding the extent of surgical resection. Differentiating PDF with a cystic component from cystic pancreatic neoplasms or cystic neuroendocrine tumors can often be challenging. However, suppose the cystic component is internally connected, forming a continuous structure rather than a cluster of separate cysts. In that case, it may reflect a dilated pancreatic duct, and PDF should be considered in the differential diagnosis. In cases with a solid component, as seen in our first case (Fig. 2), high apparent diffusion coefficient (ADC) values can help differentiate PDF from other pancreatic tumors. Nonetheless, further investigation is needed to establish these findings definitively for PDF. The continuity of cystic components within the tumor during PDF may be a strong differentiating feature that is not observed in other pancreatic tumors.

This study had some limitations. First, the number of patients was small, although this report included the largest cohort of patients who underwent PDFs with analyzable radiological findings. Second, the images were not acquired using a standardized protocol. Third, it was challenging to distinguish pathologically between the main pancreatic duct and the side branches in our two cases. Therefore, we could not definitively determine the specific involvement of the main duct or the side branches. Fourth, information regarding prognosis, post-treatment imaging changes, apparent diffusion coefficients, and standardized uptake values was limited. Future studies using this information and the sequences are required to further clarify the characteristics of PDF.

In conclusion, we have comprehensively summarized the demographic, clinical, and radiological features of PDF. PDF occurs most frequently in the pancreatic tail, and the patterns of imaging findings are classified into “solid”, “cystic”, and “solid and cystic” shapes, with solid shapes being the most common. We also found that cystic components within the tumors, suggestive of a dilated pancreatic duct, are unique imaging features of PDF. The recognition of these factors may facilitate the preoperative diagnosis of PDF.

## Data Availability

No datasets were generated or analysed during the current study.

## References

[1] Fritchie KJ, Crago AM, Van de Rijn M (2020) Soft Tissue and Bone Tumours, 5th. World Health Organization, pp 93-95

[2] Reitamo JJ, Hayry P, Nykyri E, Saxen E (1982) The desmoid tumor. I. Incidence, sex-, age- and anatomical distribution in the Finnish population. Am J Clin Pathol, 77(6):665-673 7091046 10.1093/ajcp/77.6.665

[3] Crago AM, Denton B, Salas S, et al. (2013) A prognostic nomogram for prediction of recurrence in desmoid fibromatosis. Ann Surg, 258(2):347-353 23532110 10.1097/SLA.0b013e31828c8a30 PMC4096320

[4] Salas S, Dufresne A, Bui B, et al. (2011) Prognostic factors influencing progression-free survival determined from a series of sporadic desmoid tumors: a wait-and-see policy according to tumor presentation. J Clin Oncol, 29(26):3553-3558 21844500 10.1200/JCO.2010.33.5489

[5] Nieuwenhuis MH, Casparie M, Mathus-Vliegen LM, Dekkers OM, Hogendoorn PC, Vasen HF (2011) A nation-wide study comparing sporadic and familial adenomatous polyposis-related desmoid-type fibromatoses. Int J Cancer, 129(1):256-261 20830713 10.1002/ijc.25664

[6] Clarke-Brodber AL, Hartley CP, Ahmed F, Thangaiah JJ, Tiegs-Heiden C, Hagen CE (2022) Desmoid fibromatosis involving the pancreas: A retrospective case series with clinical, cytopathologic and radiologic correlation. Ann Diagn Pathol, 60:152015 35908332 10.1016/j.anndiagpath.2022.152015

[7] Page MJ, McKenzie JE, Bossuyt PM, et al. (2021) The PRISMA 2020 statement: an updated guideline for reporting systematic reviews. Rev Esp Cardiol (Engl Ed), 74(9):790-799 34446261 10.1016/j.rec.2021.07.010

[8] Murad MH, Sultan S, Haffar S, Bazerbachi F (2018) Methodological quality and synthesis of case series and case reports. BMJ Evid Based Med, 23(2):60-63 29420178 10.1136/bmjebm-2017-110853 PMC6234235

[9] Kurokawa R, Baba A, Emile P, et al. (2022) Neuroimaging features of angiocentric glioma: A case series and systematic review. J Neuroimaging, 32(3):389-399 35201652 10.1111/jon.12983 PMC9306893

[10] Kurokawa R, Baba A, Kurokawa M, et al. (2022) Neuroimaging of astroblastomas: A case series and systematic review. Journal of Neuroimaging, 32(2):201-212 34816541 10.1111/jon.12948

[11] Kurokawa R, Baba A, Kurokawa M, et al. (2022) Neuroimaging features of diffuse hemispheric glioma, H3 G34-mutant: A case series and systematic review. J Neuroimaging, 32(1):17-27 34632671 10.1111/jon.12939

[12] Kurokawa R, Kurokawa M, Baba A, Ota Y, Moritani T, Srinivasan A (2021) Radiological features of head and neck mammary analogue secretory carcinoma: 11 new cases with a systematic review of 29 cases reported in 28 publications. Neuroradiology, 63(11):1901-1911 34427706 10.1007/s00234-021-02796-7

[13] Alghamdi HM (2021) Invasive giant pancreatic desmoid-type fibromatosis with curative resection: A case report. Int J Surg Case Rep, 86:106327 34481134 10.1016/j.ijscr.2021.106327 PMC8416951

[14] Ardakani JV, Mehrjardi AZ, Wadji MB, Saraee A (2016) A Sporadic Desmoid Tumor: an Exceptional Pancreatic Cystic-Solid Mass. Indian J Surg, 78(4):318-320 27574352 10.1007/s12262-015-1403-8 PMC4987560

[15] Bruce JM, Bradley EL, 3rd, Satchidanand SK (1996) A desmoid tumor of the pancreas. Sporadic intra-abdominal desmoids revisited. Int J Pancreatol, 19(3):197-203 8807365 10.1007/BF02787368

[16] Chen JC, Huang SC (2023) Giant desmoid fibromatosis of the pancreas. Pediatr Neonatol, 64(3):344-346 36376231 10.1016/j.pedneo.2022.08.004

[17] Dallaire DFB, Dallaire DE, Perigny DM (2018) Pancreatic desmoid tumor: A rare case with radiologic-pathologic correlation. Radiol Case Rep, 13(5):1079-1083 30228848 10.1016/j.radcr.2018.04.025 PMC6137398

[18] Gerleman R, Mortensen MB, Detlefsen S (2015) Desmoid Tumor of the Pancreas: Case Report and Review of a Rare Entity. Int J Surg Pathol, 23(7):579-584 26215223 10.1177/1066896915597752

[19] Hagiwara K, Mihara K, Aiura K, Shito M (2020) Successful outcomes after laparoscopic spleen-preserving pancreatic resection for a desmoid tumor: A case report. Int J Surg Case Rep, 74:117-119 32836204 10.1016/j.ijscr.2020.07.076 PMC7452466

[20] Jafri SF, Obaisi O, Vergara GG, et al. (2017) Desmoid type fibromatosis: A case report with an unusual etiology. World J Gastrointest Oncol, 9(9):385-389 28979721 10.4251/wjgo.v9.i9.385 PMC5605339

[21] Jia C, Tian B, Dai C, Wang X, Bu X, Xu F (2014) Idiopathic desmoid-type fibromatosis of the pancreatic head: case report and literature review. World J Surg Oncol, 12:103 24755337 10.1186/1477-7819-12-103 PMC4032157

[22] Khanna K, Mofakham FA, Gandhi D, Jain N (2020) Desmoid fibromatosis of the pancreas--A case report with radiologic-pathologic correlation. Radiol Case Rep, 15(11):2324-2328 32994833 10.1016/j.radcr.2020.08.061 PMC7501420

[23] Lee KC, Lee J, Kim BH, Kim KA, Park CM (2018) Desmoid-type fibromatosis mimicking cystic retroperitoneal mass: case report and literature review. BMC Med Imaging, 18(1):29 30223791 10.1186/s12880-018-0265-5 PMC6142631

[24] Li T, Gao YC, Chen B, Zhang ZL (2019) Aggressive Fibromatosis of the Pancreas: A Rare Cause of Incomplete Duodenal Obstruction. Pancreas, 48(3):e16-e17 30855430 10.1097/MPA.0000000000001246

[25] Litchinko A, Brasset C, Tihy M, Amram ML, Ris F (2022) Large Desmoid Tumor of the Pancreas: A Report of a Rare Case and Review of the Literature. Am J Case Rep, 23:e937324 36378606 10.12659/AJCR.937324 PMC9676065

[26] Meyer A, Szajnbok P, Koszka AJM, Pezzutti D, Segatelli V, Monteiro J, Jr. (2021) A rare sporadic pancreatic desmoid fibromatosis with splenic vein invasion diagnosed by CT scan-guided core needle biopsy: a case report with possible differential diagnosis from metastatic colorectal or renal cancer. J Surg Case Rep, 2021(6):rjab257 34194727 10.1093/jscr/rjab257 PMC8238400

[27] Mourra N, Ghorra C, Arrive L (2015) An Unusual Solid and Cystic Pancreatic Tumor in a 20-Year-Old Woman. Desmoid Tumor: Fibromatosis. Gastroenterology, 149(3):e5-6 26226587 10.1053/j.gastro.2014.12.056

[28] Nursal TZ, Abbasoglu O (2003) Sporadic hereditary pancreatic desmoid tumor: a new entity? J Clin Gastroenterol, 37(2):186-188 12869894 10.1097/00004836-200308000-00019

[29] Park CG, Lee YN, Kim WY (2021) Desmoid type fibromatosis of the distal pancreas: A case report. Ann Hepatobiliary Pancreat Surg, 25(2):276-282 34053932 10.14701/ahbps.2021.25.2.276 PMC8180399

[30] Pho LN, Coffin CM, Burt RW (2005) Abdominal desmoid in familial adenomatous polyposis presenting as a pancreatic cystic lesion. Fam Cancer, 4(2):135-138 15951964 10.1007/s10689-004-1923-z

[31] Polistina F, Costantin G, D’Amore E, Ambrosino G (2010) Sporadic, nontrauma-related, desmoid tumor of the pancreas: a rare disease-case report and literature review. Case Rep Med, 2010:272760 20300597 10.1155/2010/272760 PMC2838224

[32] Shayesteh S, Salimian KJ, Fouladi DF, Blanco A, Chu LC, Fishman EK (2020) Pancreatic cystic desmoid tumor following metastatic colon cancer surgery: A case report. Radiol Case Rep, 15(11):2063-2066 32944101 10.1016/j.radcr.2020.08.013 PMC7481488

[33] Slowik-Moczydlowska Z, Rogulski R, Piotrowska A, Maldyk J, Kluge P, Kaminski A (2015) Desmoid tumor of the pancreas: a case report. J Med Case Rep, 9:104 25943401 10.1186/s13256-015-0591-y PMC4437747

[34] Torres JC, Xin C (2018) An unusual finding in a desmoid-type fibromatosis of the pancreas: a case report and review of the literature. J Med Case Rep, 12(1):123 29751773 10.1186/s13256-018-1635-x PMC5948877

[35] Tsukamoto Y, Imakita M, Nishitani A, Ito T, Izukura M, Hirota S (2016) Pancreatic desmoid-type fibromatosis with beta-catenin gene mutation-Report of a case and review of the literature. Pathol Res Pract, 212(5):484-489 26907785 10.1016/j.prp.2016.02.012

[36] Wang YC, Wong JU (2016) Complete remission of pancreatic head desmoid tumor treated by COX-2 inhibitor-a case report. World J Surg Oncol, 14(1):190 27450394 10.1186/s12957-016-0944-z PMC4957301

[37] Weiss ES, Burkart AL, Yeo CJ (2006) Fibromatosis of the remnant pancreas after pylorus-preserving pancreaticoduodenectomy. J Gastrointest Surg, 10(5):679-688 16773761 10.1016/j.gassur.2005.09.029

[38] Xu B, Zhu LH, Wu JG, Wang XF, Matro E, Ni JJ (2013) Pancreatic solid cystic desmoid tumor: case report and literature review. World J Gastroenterol, 19(46):8793-8798 24379602 10.3748/wjg.v19.i46.8793 PMC3870530

[39] Xuesong D (2020) Pancreatic desmoid tumor with unusual imaging features. Pancreatology, 20(5):1015-1016 32694006 10.1016/j.pan.2020.05.014

[40] Huang K, Wang CM, Chen JG, et al. (2014) Prognostic factors influencing event-free survival and treatments in desmoid-type fibromatosis: analysis from a large institution. Am J Surg, 207(6):847-854 24119719 10.1016/j.amjsurg.2013.08.007

[41] Zeng WG, Zhou ZX, Liang JW, et al. (2014) Prognostic factors for desmoid tumor: a surgical series of 233 patients at a single institution. Tumour Biol, 35(8):7513-7521 24789435 10.1007/s13277-014-2002-1

[42] Bhattacharya B, Dilworth HP, Iacobuzio-Donahue C, et al. (2005) Nuclear beta-catenin expression distinguishes deep fibromatosis from other benign and malignant fibroblastic and myofibroblastic lesions. Am J Surg Pathol, 29(5):653-659 15832090 10.1097/01.pas.0000157938.95785.da

[43] Askan G, Basturk O (2022) Mesenchymal Tumors Involving the Pancreas: A Clinicopathologic Analysis and Review of the Literature. Turk Patoloji Derg, 38(1):46-53 35001360 10.5146/tjpath.2022.01567 PMC9999697

[44] Miettinen M, Lasota J (2006) Gastrointestinal stromal tumors: review on morphology, molecular pathology, prognosis, and differential diagnosis. Arch Pathol Lab Med, 130(10):1466-1478 17090188 10.5858/2006-130-1466-GSTROM

